# Hyperdominant Left Circumflex Artery Challenging Conventional Paradigms and Current Guideline Recommendations: A Comprehensive Literature Review and Case Series

**DOI:** 10.1002/ccr3.73801

**Published:** 2026-10-29

**Authors:** Amirmahdi Mohammadi, Mohammad Sadegh‐Vashveshady

**Affiliations:** ^1^ Department of Cardiovascular Surgery, School of Medicine Semnan University of Medical Sciences Semnan Iran; ^2^ Cardiac Electrophysiology Research Center Rajaie Cardiovascular Institute Tehran Iran; ^3^ Department of Cardiology, School of Medicine Semnan University of Medical Sciences Semnan Iran

**Keywords:** acute coronary syndrome, case series, guidelines, isolated posterior myocardial infarction, left coronary dominant, literature review, ST‐elevation myocardial infarction

## Abstract

Despite its rarity in the general population, left dominant coronary circulation with a hyperdominant left circumflex artery (LCx) has been associated with a more severe risk for ischemic and coronary artery disease. The concealment of an isolated posterior myocardial infarction (IPMI) on a standard 12‐lead ECG among these patients with distinctive coronary anatomy can be concerning, especially when clinicians become trapped within conventional guideline‐based diagnostic criteria, a circumstance that may ultimately be linked to a poorer prognosis. We present two cases that posed unique diagnostic challenges in this specific anatomical context. Case 1: A 63‐year‐old man presented to the emergency department (ED) with typical anginal chest pain and severe fatigue. Although the patient was initially diagnosed with non–ST‐elevation myocardial infarction (NSTEMI), eventually a catastrophic occlusion of the left posterior descending artery (PDA) in the setting of a hyperdominant LCx was seen during coronary angiography (CAG). Case 2: A 69‐year‐old man arrived at the ED with typical angina, described as pressure‐like. Although the serial 12‐lead ECGs were completely normal, given the clinical context, the patient underwent an immediate invasive strategy in < 2 h. CAG revealed complete occlusion of the ostio‐proximal segment of the left PDA. In both cases, hyperdominant LCx anatomy created a diagnostic blind spot within the conventional STEMI/NSTEMI paradigm, because the infarct territory was insufficiently captured by standard ECG criteria and failed to trigger guideline‐based use of posterior leads or adjunctive imaging, thereby obscuring the diagnosis of IPMI. These cases warn clinicians about the diagnostic complexity of IPMI in a hyperdominant LCx setting. In such cases, ECG findings can be ambiguous or even completely absent, and the clinical presentation may not meet current unclear guideline‐based indications for adjunctive diagnostic assessment, leading to delayed recognition of high‐risk ischemia, particularly IPMI.

## Key Clinical Message

1. In all NSTEMI patients with ambiguous symptoms and nondiagnostic ECGs, the routine acquisition of posterior chest leads should no longer be overlooked.
2. In the presence of a hyperdominant‐LCx, occlusion of the artery or its branches may result in extensive yet clinically silent infarctions that masquerade as typical NSTEMI.

## Graphical abstract

Summary of the patient's presentation, diagnostic evaluation, treatment, and follow‐up.

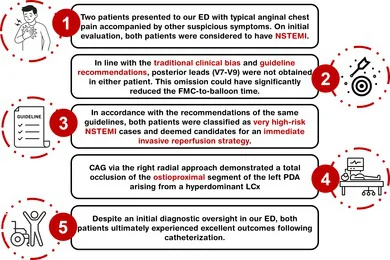

**Abbreviations**
ACS: acute coronary syndrome
CAG: coronary angiography
IPMI: isolated posterior myocardial infarction
LCx: left circumflex artery
MACE: major adverse cardiovascular events
NSTEMI: Non–ST‐elevation myocardial infarction
PCI: percutaneous coronary intervention
PDA: posterior descending artery
RCA: right coronary artery
STD: ST‐segment depression
STE: ST‐segment elevation

## 1 Introduction

Coronary artery dominance is defined by the artery that gives rise to the PDA and posterolateral vessel (PLV), determining the primary source of perfusion of the inferior/posterior left ventricular myocardial territories and posterior portion of the interventricular septum [1, 2]. Left coronary dominance is observed in approximately one‐tenth of the population, yet the presence of a hyperdominant‐LCx is an extremely rare anatomical variant [1, 3]. To date, in the lack of a universally accepted definition and given the heterogeneity of descriptions across the available literature, coronary hyperdominance has generally been used to describe a large coronary artery with an extended distribution that provides blood flow to substantial myocardial territories normally perfused by the contralateral coronary artery [2, 3]. Of note, an unusually long and large‐caliber epicardial vessel does not per se define coronary hyperdominance; for instance, a Type 3 (wrap‐around) LAD extends past the apex to perfuse at least a quarter of the inferior wall in up to 35% of the population without replacing the PDA or conferring a hyperdominant circulation [4]. Although the anatomical complexity of coronary hyperdominance is ultimately recognized through angiographic evaluation, it is associated with a distinct susceptibility and early‐phase pattern of clinical manifestations in relation to specific myocardial infarction subtypes [1, 5]. Thereby providing a setting for extensive yet concealed ischemia [1, 5, 6].

As one of the most clinically under‐recognized forms of ischemia, IPMI occurs with remarkably low frequency among posterior wall involvements [6, 7]. This rarity is typically associated with heightened diagnostic difficulties during initial evaluation, ultimately leading to adverse clinical outcomes [6]. This challenge may become even more exacerbated when IPMI occurs in the setting of a hyperdominant‐LCx, where the potential for clinical masking and serious outcomes may be further compounded.

Herein, we describe two diagnostically challenging cases of IPMI in the setting of a hyperdominant‐LCx, resulting from ostio‐proximal obstruction of the left PDA, a diagnosis that remained undetected on the standard 12‐lead ECG. Consequently, in line with the conventional interpretation of the routine 12‐lead ECG by emergency physicians and its perceived alignment with guideline criteria, neither posterior leads nor additional imaging modalities were initially obtained in either patient. Nevertheless, adherence to the same guideline‐recommended prompt invasive strategy ultimately proved lifesaving in both cases.

## 2 Case Presentation 1

A 63‐year‐old man was referred to our hospital with angina, diaphoresis, and worsening fatigue. Despite daily use of aspirin, he had no known history of coronary artery disease (CAD). He also denied having diabetes mellitus or a history of smoking.

Hemodynamically stable on admission with an unremarkable cardiac exam, the patient presented with lateral ST‐segment changes and elevated troponin‐T. Serial ECGs demonstrated persistent subtle lateral ST‐segment depression (< 0.5 mm in V5–V6; Figure 1). Initial labs were notable for HbA1c 5.7% and LDL‐C 142 mg/dL. Despite treatment with standard cardiovascular medications at the referring hospital, refractory angina accompanied by a rise in troponin T (8160 ng/L) led to the patient being classified as a very high‐risk NSTEMI profile. Furthermore, he was identified as a candidate for an immediate invasive strategy with CAG within 2 h (Table 1). The angiogram showed acute thrombotic occlusion at the ostium of the left PDA in the setting of a left‐dominant coronary circulation, resulting in extensive IPMI (Figure 2A,B). The hyperdominant‐LCx, which was widely patent and free of significant stenosis, supplied myocardial territories that are typically perfused by the right coronary artery (RCA) (Figure 2C). Additionally, the left anterior descending artery (LAD) showed two moderate atherosclerotic plaques (~50% stenosis) at the proximal and middle segments, which could potentially contribute to the subtle, non‐dynamic, STD observed in the lateral precordial leads (Figure 2C). The RCA revealed multiple proximal concentric stenoses; however, as it was not the infarct‐related artery, no emergent percutaneous coronary intervention (PCI) was performed (Figure 2D).

**FIGURE 1:**
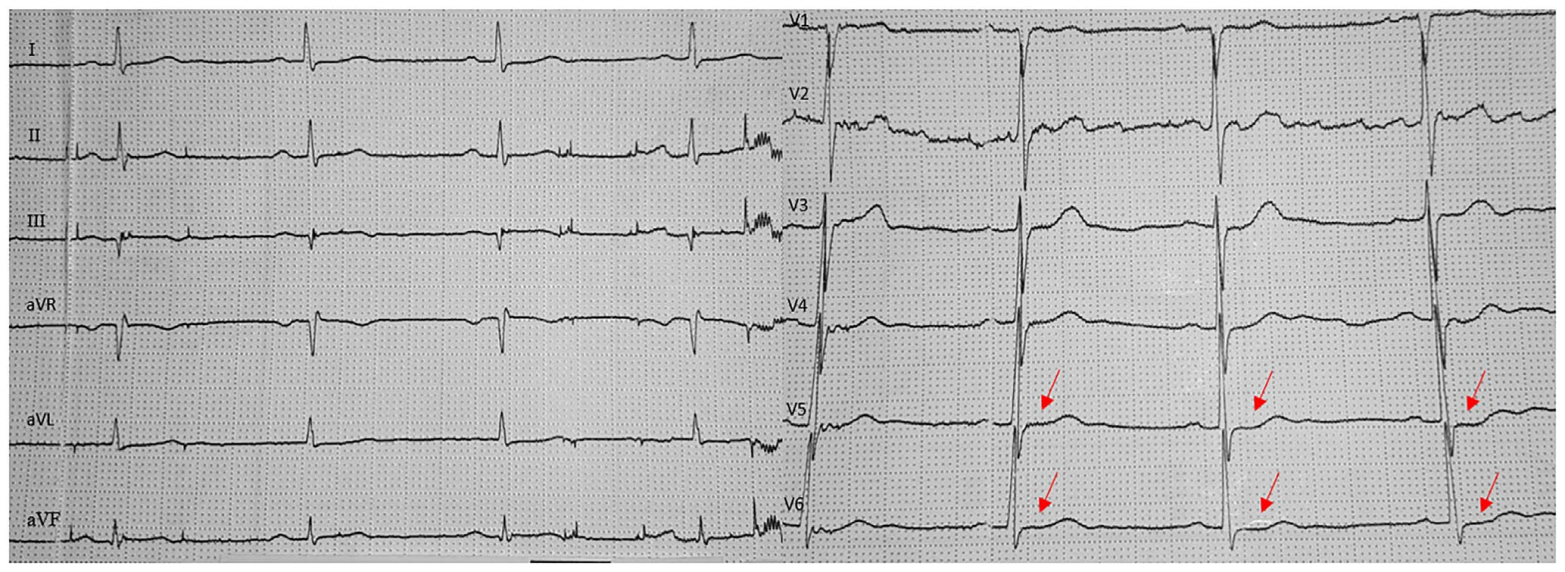
ECG of IPMI mimicking NSTEMI in the first patient. The 12‐lead ECG of the patient at our hospital showed transient subtle STD in lateral precordial leads (thin red arrows).

**TABLE 1:** What do the current clinical guidelines recommend regarding the selection of invasive strategies in patients with NSTE‐ACS? Similarities and differences.

Risk categories	Risk criteria	Recommended strategy	FMC to CAG/PCI time	Class strength for strategy and timing
Unstable (very‐high‐risk patient)	1. Recurrent or ongoing angina refractory to medical therapy^A,B^ 2. Hemodynamic instability or CS^A,B^ 3. Signs or symptoms of acute HF presumed secondary to ischemia (e.g., new/worsening MR or acute pulmonary edema)^A^ 4. Life‐threatening arrhythmias or electrical instability (e.g., VF and VT)^A,B^ 5. Recurrent dynamic ECG changes suggestive of ischemia (e.g., intermittent STE)^B^ 6. Cardiac arrest after presentation^B^ 7. Mechanical complications^B^	Immediate invasive strategy	< 2 h	Invasive strategy (Class 1)/timing (Class 1)
High risk (NSTEMI)	1. Confirmed diagnosis of NSTEMI based on ECG hs‐cTn algorithms^B^ 2. GRACE risk score > 140^A,B^ 3. Steeply rising TN values on serial measurement despite medical reperfusion therapy^A,B^ 4. Dynamic ST‐segment or T wave change^A,B^ 5. Transient STE^B^	Inpatient invasive strategy	< 24 h	Invasive strategy (Class 1)/timing (Class 2a)
Intermediate risk (NSTE‐ACS)	1. GRACE risk score 109–140^A^ 2. No evidence of ongoing ischemia symptoms^A^ 3. Stable or reducing Tn levels^A^	Inpatient invasive strategy	< 72 h	Invasive strategy (Class 1)/timing (Class 2a)
Lower risk (NSTE‐ACS)	1. Grace risk score < 109^A^ 2. TIMI risk score < 2^A^ 3. No evidence of ongoing ischemia symptoms^A^ 4. Tn < 99th percentile^A^ 5. No dynamic ST‐segment changes^A^	Inpatient invasive strategy/selective invasive strategy (in patients who are considered to have a low index of suspicion for NSTE‐ACS without high or very‐high risk features, management should be guided according to ESC guidelines for CCS)	< 72 h (for inpatient invasive strategy)/during hospitalization (for selective invasive strategy)	Invasive strategy (Class 1)/timing (Class 2a)

*Note:* “A” denotes evidence and recommendations derived from the 2025 ACC/AHA/ACEP/NAEMSP/SCAI Clinical Practice Guideline on acute coronary syndromes. “B” denotes recommendations supported by the 2023 ESC Guidelines for the Management of Acute Coronary Syndromes. “A, B” denotes recommendations endorsed by both the 2025 ACC/AHA/ACEP/NAEMSP/SCAI and the 2023 ESC guidelines. The 2023 ESC guidelines for the management of acute coronary syndromes do not provide clearly defined criteria for intermediate‐risk or lower‐risk patients. Instead, patients who do not meet the criteria for high‐risk or very high‐risk categories are collectively classified as non–high‐risk.

Abbreviations: CCS, chronic coronary syndrome; CS, cardiogenic shock; GRACE, global risk of acute coronary events; HF, heart failure; MR, mitral regurgitation; NSTE‐ACS, non ST‐segment elevation acute coronary syndrome; TIMI, thrombolysis in myocardial infraction; VF, ventricular fibrillation; VT, ventricular tachycardia.

**FIGURE 2:**
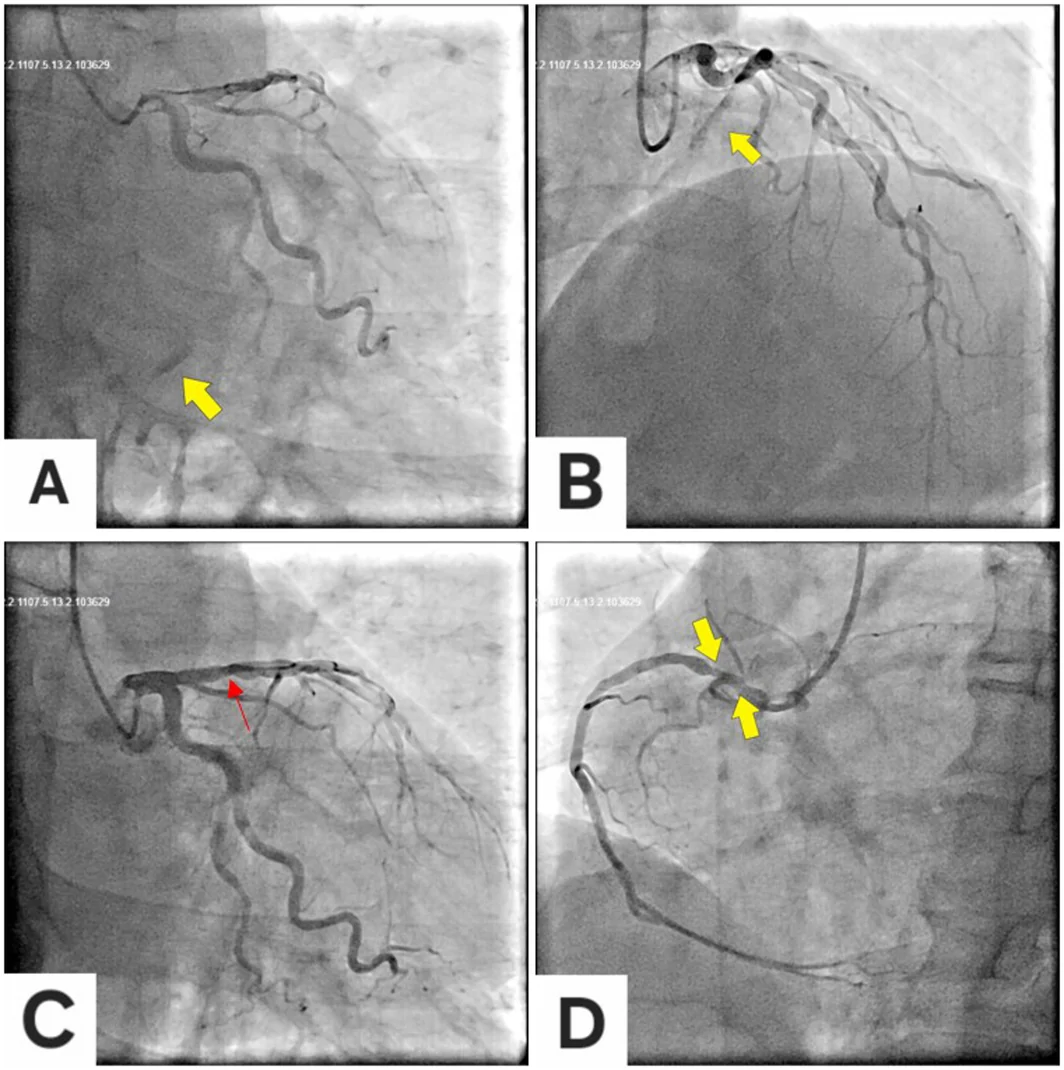
Coronary angiography findings in the first patient, immediate CAG revealed: (A) RAO caudal view and (B) AP cranial view showing a large thrombotic occlusion (thick yellow arrows) in the left PDA following contrast injection. (C) LAO cranial view showing a markedly enlarged hyperdominant‐LCx with a disproportionately large caliber relative to the other coronary arteries and an extended course reaching and covering the left ventricular apex, as well as a moderate atherosclerotic plaque (thin red arrow) in the proximal segment of the LAD. (D) LAO view showing a nondominant right coronary artery with multiple significant ostioproximal stenoses. AP, anteroposterior; CAG, coronary angiography; LAD, left anterior descending artery; LAO, left anterior oblique; RAO, right anterior oblique.

The left coronary was engaged with a 6F XB 3.0 guide catheter. After crossing the occluded PDA ostium with a 0.014‐in. guidewire, we performed pre‐dilation using a 2.0 × 10 mm balloon. Due to small distal caliber confirmed post‐intracoronary nitroglycerin, stenting was deferred. Balloon‐only PCI successfully restored TIMI 3 flow (Figure 3A–E). Given that the lesion was located at the ostium of a small‐caliber vessel and full distal flow was achieved following balloon dilation, the consensus of the heart team was to defer placement of a drug‐eluting stent (DES).

**FIGURE 3:**
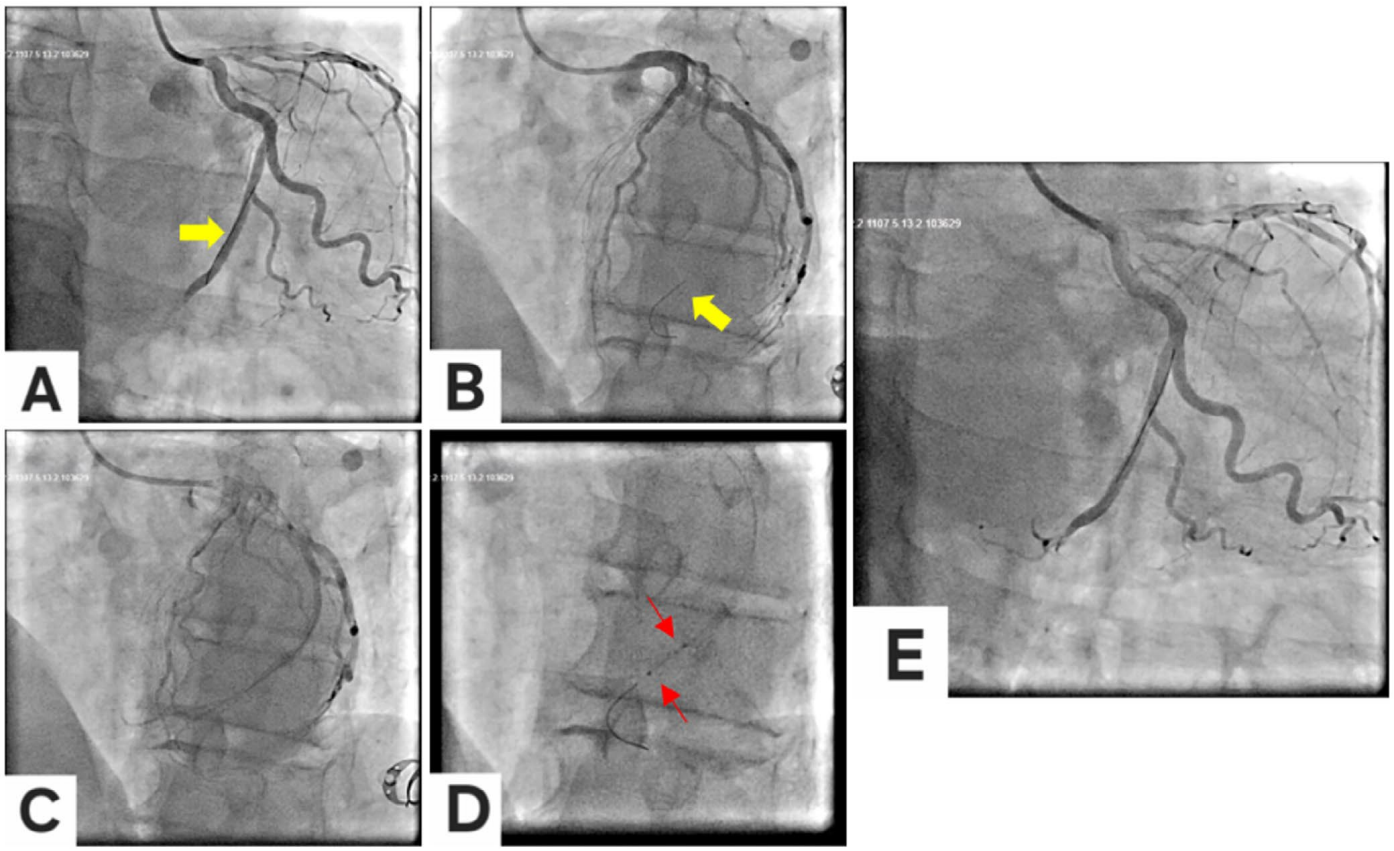
Percutaneous coronary intervention findings in the first patient. During PCI it was observed that (A) RAO caudal view and (B) LAO cranial view demonstrating restoration of antegrade flow in the left PDA following guidewire crossing (thick yellow arrow). (C–E) Balloon angioplasty (red arrows) of the proximal PDA was performed, resulting in improved luminal diameter and enhanced distal flow. LAO, left anterior oblique; PCI, percutaneous coronary intervention; RAO, right anterior oblique.

## 3 Case Presentation 2

The second case involved a 69‐year‐old man who presented to our ED with typical angina radiating to the neck, back, and left arm along with diaphoresis. The pain began at rest approximately 2 h before presentation and was characterized as a pressure‐type chest discomfort. He had a history of hyperlipidemia and denied a history of diabetes mellitus, tobacco use, or prior cardiovascular disease.

On physical examination, his vital signs were as follows: blood pressure 119/86 mmHg, heart rate 85 bpm, respiratory rate 17 breaths/min, and oxygen saturation 94% on room air. Cardiac auscultation revealed normal S_1_ and S_2_ heart sounds, with systolic murmurs consistent with moderate mitral and tricuspid regurgitation confirmed on prior echocardiography.

The serial 12‐lead ECGs did not show ST‐segment elevation (STE) or acute T‐wave changes; however, initial laboratory evaluations revealed an HbA1c level of 5.6%, consistent with the absence of diabetes mellitus, and an elevated LDL‐C level of 178 mg/dL, consistent with his known history of hyperlipidemia (Figure 4). Nevertheless, in light of the patient's clinical context, he was placed on continuous cardiac monitoring in the ED. Significant elevation of troponin‐T values (11,190 ng/L) prompted initiation of dual antiplatelet therapy (DAPT) with aspirin and clopidogrel, anticoagulation with low molecular weight heparin, along with high‐intensity atorvastatin. Despite the absence of any dynamic ECG changes during this time, the presence of refractory angina and a continued rise in troponin‐T levels categorized him as a very‐high‐risk NSTEMI, prompting an immediate invasive strategy (Table 1).

**FIGURE 4:**
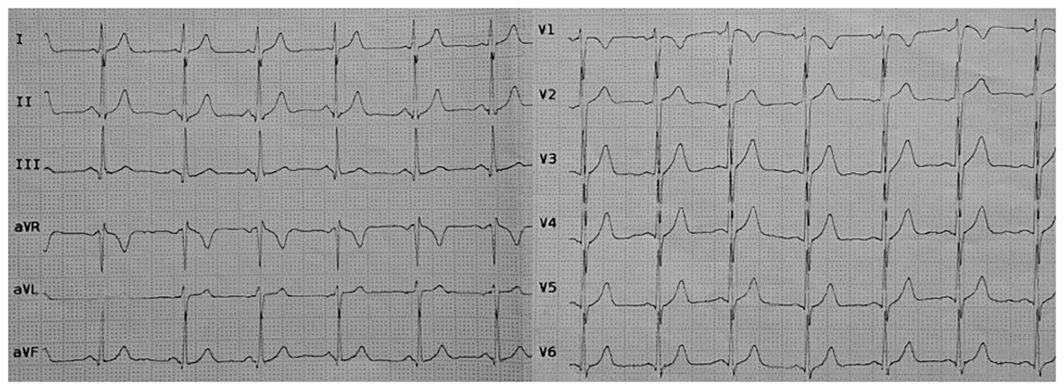
12‐Lead ECG in the second patient. One of the initial 12‐lead ECG in the second patient was entirely normal. PDA, posterior descending artery.

The patient underwent emergent cardiac catheterization within 90 min. CAG revealed a total occlusion of the ostio‐proximal left PDA within a hyperdominant left circumflex system (Figure 5A). The LAD exhibited < 20% proximal stenosis, while the first obtuse marginal (OM1) branch showed significant proximal concentric stenosis (Figure 5B,C). The RCA was diminutive, reflecting a left‐dominant coronary anatomy (Figure 5D). Because the culprit lesion was presented in the left PDA and the OM1 lesion was an incidental but stable finding, first PCI was limited to the infarct‐related artery to minimize procedural complexity and contrast load.

**FIGURE 5:**
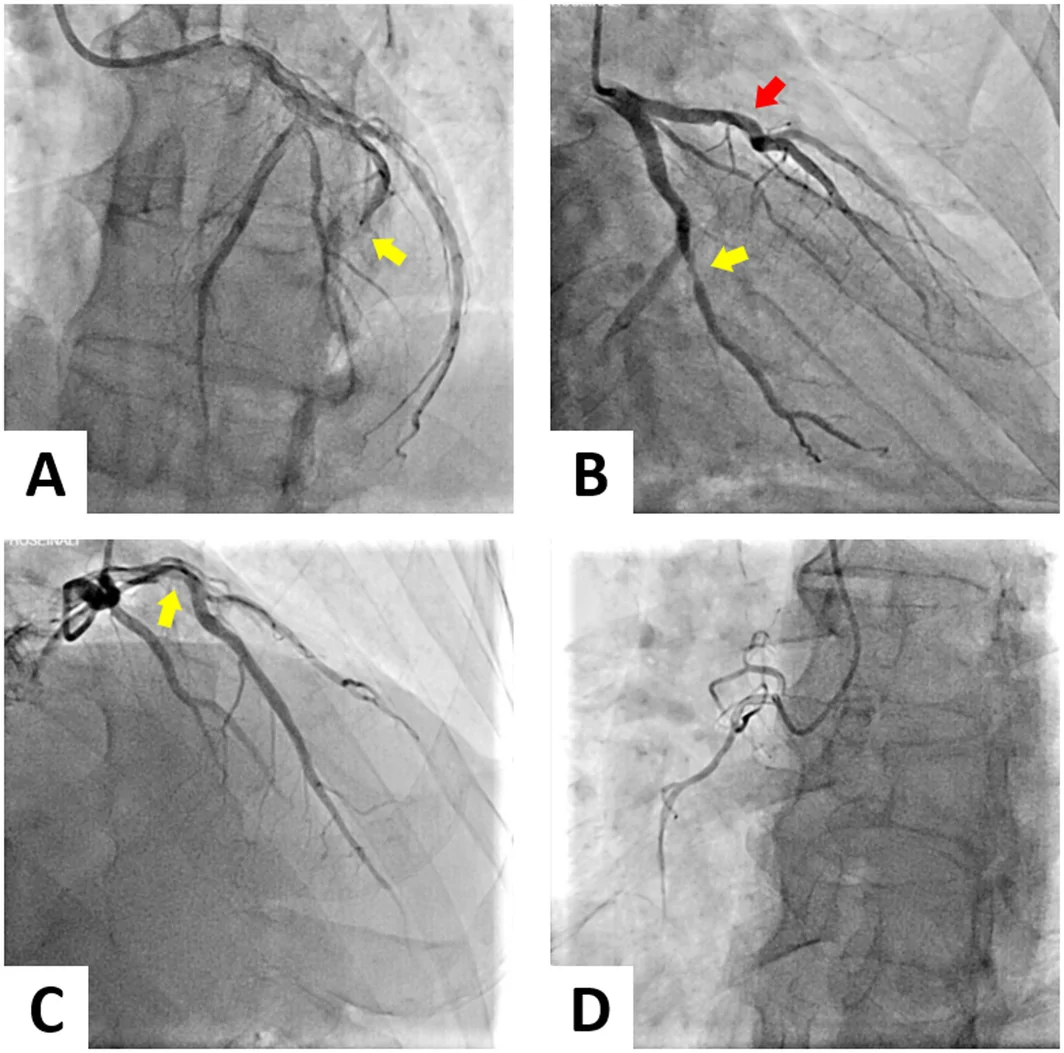
Coronary angiography findings in the second patient. CAG revealed: (A) LAO cranial view showing the proximal portion of the large LCx artery; its ostium is well visualized, and a total occlusion (complete flow interruption) is present at the origin of the left PDA following contrast injection (yellow thick arrow). (B) RAO caudal view showing a patent left main coronary artery without significant stenosis; however, a concentric ostioproximal stenosis (yellow thick arrow) is evident in the OM1. The proximal segment of the LAD artery exhibits mild plaque extension (B: red thick and C: red yellow arrow). RAO cranial view demonstrating a total eccentric occlusion (thick yellow arrows) in its distal segment involving the left PDA. (D) LAO view depicting a diminutive RCA without significant stenosis or atherosclerotic plaque. LAD, left anterior descending artery; LAO, left anterior oblique; LCx, left circumflex artery; OM1, first obtuse margin branch; RAO, right anterior oblique.

A guiding catheter and a 0.014‐in. coronary guidewire, similar to those used in the first patient, were advanced across the lesion. Predilatation was performed using a 2.0 × 12 mm balloon. Implantation of a 2.50 × 28 mm DES in the residual lesion resulted in complete distal reperfusion of the left PDA, with TIMI flow improving from grade 0 to 3 (Figure 6A–G).

**FIGURE 6:**
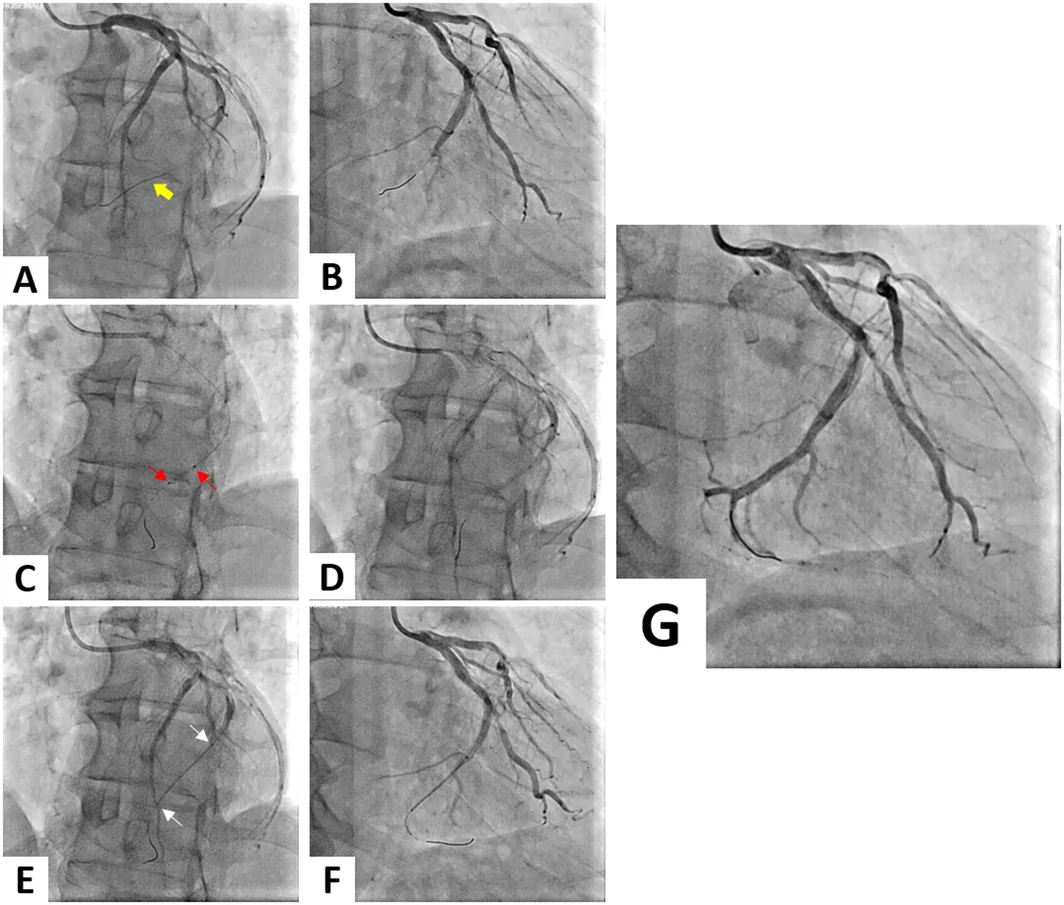
Percutaneous coronary intervention findings in the second patient. During PCI, (A) LAO cranial view and (B) RAO caudal view demonstrate successful coaxial crossing of the guidewire through the LCx artery into the left PDA (yellow thick arrow). (C) LAO cranial view demonstrating balloon angioplasty (red thin arrows) of the proximal segment of the left PDA. (D) after balloon dilatation showing improve luminal diameter and enhanced distal flow. (E–G) LAO cranial and RAO caudal views after successful stent deployment in the PDA (white thin arrows), confirming successful implantation and restoration of TIMI 3 flow. Owing to its markedly large caliber relative to the other epicardial coronary arteries and its extended distribution into myocardial territories normally supplied by the RCA, the LCx was classified hyperdominant. LAO, left anterior oblique; LCx, left circumflex artery; PDA, posterior descending artery; RAO, right anterior oblique.

Post‐PCI echocardiography was unremarkable in both patients, showing no segmental wall‐motion abnormalities, significant valvular dysfunction, or intracardiac thrombus. Following a 2‐day hospitalization, patients were discharged on GDMT. DAPT (aspirin and ticagrelor) was initiated and successfully de‐escalated to aspirin and clopidogrel at 4 weeks, continuing for a total of 12 months [8, 9]. This decision was driven by an individualized balance of bleeding risk versus ischemic benefit, corroborated by local medication shortages and an established regional clinical pattern of reduced compliance among elderly patients, presumably secondary to ticagrelor‐related dyspnea, frequently reported in our center. Each month later, PCI of RCA was recommended for the first patient, but he chose not to be hospitalized. Similarly, PCI of the first obtuse marginal (OM1) branch was advised for the second one, who also declined admission. As of this writing, the procedures have been well tolerated, and the patients remain asymptomatic.

## 4 Discussion and Literature Review

Since Monroe Schlesinger first established the concept of coronary dominance in 1940 through postmortem radiographic autopsy evaluations, advancements in cardiac imaging modalities have clarified that roughly 85% of individuals are right dominant, 8% are left‐dominant, and 7% display a codominant anatomy [1, 10] (Table 2). Despite the fact that left‐dominant coronary circulation is observed in only 8%–10% of the population, it has been associated with several important issues, such as increased risk of ischemic myocardial events [1]. Due to the combination of higher blood flow volume and the longer anatomical course of vessels originating from the left main coronary artery [1]. Although in the hyperdominant‐LCx setting, the predominant coronary flow is delivered through this vessel, potentially altering coronary flow dynamics and predisposing atherosclerotic changes, the LCx or its branches are seldom identified as the culprit vessel in PMI [1, 6, 11]. This phenomenon becomes particularly apparent given that all documented IPMI cases to date have been associated with occlusions within RCA branches. In situations where the LCx or its branches serve the culprit vessel in STEMI, correct interpretation of ECG manifestations becomes challenging [12]. A complexity may become more pronounced in association with hyperdominant‐LCx [1, 3, 12]. At this point, ECG changes may be overlooked or misinterpreted as NSTEMI, notably in IPMI, which carry a substantially greater diagnostic challenge. In addition, posterior wall ischemia may present with subtle or unusual manifestations in standard 12‐lead ECGs, the diagnostic complexity of LCx involvement extends beyond static ST‐segment scrutiny; rather, extrasystole‐triggered changes may transiently unmask an otherwise silent LCx‐related occlusion, even when sinus beats remain nondiagnostic [13]. This diagnostic challenge is further amplified for less experienced clinicians. These findings indicate the necessity for a more comprehensive diagnostic evaluation, including the use of posterior leads (V7–V9) and multimodal imaging techniques [6, 8, 9, 11, 12, 14]. Nevertheless, selective placement of additional leads based on detailed coronary anatomy adds little diagnostic value for the early detection of concealed STEMI, since definitive delineation of coronary dominance is usually achieved during the ultimate diagnostic step [8, 9].

**TABLE 2:** Literature‐based anatomical variants of epicardial coronary dominance. While other extremely rare coronary anomalies and atypical branching patterns have been occasionally described, this table summarizes the most commonly recognized and frequently reported anatomical variants in the literature.

Dominance category	Subtype/Pattern	Anatomical definition and vessel course	PDA and PLV status	Status of remaining vessels (reciprocal changes)	Approximate prevalence
1. Right dominant (~85%–88%)	Normal right dominant	The RCA supplies the posterior/inferior interventricular groove and posterior portion of the interventricular septum; the LAD and LCx perfuse their standard anterior and lateral territories.	PDA: from RCA; PLV: from RCA (≥ 1 major branch)	The LAD and LCx are typically of normal length and caliber	~80%–85%
	Hyperdominant‐RCA	The RCA is markedly enlarged and elongated; after reaching the crux and giving off the PDA/PLV, it continues into the left AV groove, supplying the posterolateral and lateral LV wall.	PDA: from RCA; PLV: entirely from RCA (multiple major branches)	Hypoplastic, markedly shortened, rudimentary, or in extreme cases, entirely absent (congenital LCx agenesis) with complete lateral territory annexation by the RCA	Extremely rare (~0.0008%)
2. Left dominant (~8%–10%)	Classic left dominant	The LCx continues along the left AV groove to the crux and gives rise to the PDA and PLV branches; the RCA is nondominant.	PDA: from the distal LCx; PLV: from the LCx	Small/short RCA terminating before the crux (typically at the acute marginal branch of the RV)	~8%–10%
	Hyperdominant‐LCx	The LCx supplies not only the PDA and all PLV branches but extends beyond the crux into the right AV groove, perfusing the posterior and marginal RV branches.	PDA: from LCx; PLV: entirely from LCx	Severely hypoplastic RCA, or in some instances, complete absence (congenital agenesis) of the RCA	Extremely rare
	Hyperdominant‐LAD	The LAD is extremely long, extends along the entire posterior interventricular groove toward the crux, effectively assuming the role of the main PDA and its inferoseptal branches.	PDA: typically, direct continuation of the LAD; PLV: typically, from LCx or terminal distal LAD branches (in select cases, intercoronary communication may establish shared PDA/PLV territory perfusion)	Both RCA and LCx are hypoplastic/small (the LAD has monopolized the coronary supply)	Extremely rare
3. Codominant/balanced	Classic codominant	Inferior and posterior perfusion is shared between the right and left systems, with complete symmetry at the crux.	PDA: from RCA; PLV: from LCx	Both RCA and LCx are of adequate and balanced caliber	~7%
	Dual/parallel PDA codominance	Two separate, parallel PDAs are present, one arising from the RCA and the other from the LCx (or the distal LAD), both coursing within the posterior interventricular groove.	PDA 1: from RCA; PDA 2: from LCx (or LAD)	Both the right and left coronary systems contribute to the posterior septal supply	Extremely rare

Abbreviations: AV, atrioventricular; LAD, left anterior descending artery; LCx, left circumflex artery; LV, left ventricle/left ventricular; PDA, posterior descending artery; PLV, posterolateral ventricular branch; RCA, right coronary artery; RV, right ventricle/right ventricular.

Consequently, a broader use of additional posterior leads may be warranted to minimize the risk of missed IPMI in such dominance pattern. However, the routine utilization of these leads is currently not recommended for all patients initially presenting with NSTEMI [8, 9]. In line with current ACS guideline criteria and prevailing clinical practice, posterior leads (V7–V9) are primarily obtained in patients with STD ≥ 0.5 mm in leads V1–V3, a pattern that is traditionally regarded as a characteristic ECG pattern of PMI [8, 9]. Nonetheless, far fewer clinical indications have been defined regarding the use of imaging modalities in these settings [8]. This created a diagnostic blind spot, potentially resulting in rare ischemic events, particularly IPMIs, to remain undetected. In addition, the intrinsic diagnostic obscurity of IPMI, when coupled with the inherently silent ischemic nature of the LCx territory, may ultimately lead to fatal outcomes.

In both cases, a critical diagnostic trap was the omission of additional leads prior to transfer to the cath‐lab. This diagnostic oversight, which reflects the routine traditional interpretation of their ECGs and the unclear guideline‐based criteria for the use of additional diagnostic leads, obscured the timely detection of IPMI in the ED, as only transient subtle STD in leads V5–V6 was observed in the first case, and no diagnostic changes were present in leads V1–V3 in either patient. We hypothesize that this distinctive coronary dominance pattern and the specific site of occlusion led to the significant diagnostic difficulties in identifying IPMI in our patients. Within up‐to‐date literature, acute occlusions involving a hyperdominant‐LCx system represent an infrequent yet profoundly consequential clinical entity. Oh et al. documented a 57‐year‐old with congenital RCA agenesis presenting with cardiogenic shock. ECG findings of aVR‐STE, precordial STD (V2–V4), and posterior STE (V7–V9) prompted a diagnosis of acute myocardial infarction and emergent CAG [15]. Angiography revealed proximal hyperdominant‐LCx occlusion and RCA agenesis. Post‐PCI (TIMI 3 flow), the vessel showed two major branches: one following standard LCx distribution, and the second, a retrograde course along the atrioventricular groove supplying the entire RCA territory. CCTA and aortography confirmed the diagnosis [15]. Khan et al. reported a case of inferior STEMI due to hyperdominant‐LCx occlusion in a patient with congenital RCA agenesis. The hyperdominant‐LCx, which supplied the RCA territory, manifested as STE in leads II, III, and aVF with reciprocal changes, necessitating emergent primary PCI [16]. Matte et al. described an 80‐year‐old man with congenital absence of the RCA ostium and a chronically occluded LAD who presented with an overt infero‐postero‐lateral STEMI. CAG revealed acute thrombotic subocclusion in the proximal‐to‐mid segments of a hyperdominant‐LCx that served as the sole remaining vessel supplying the entire coronary circulation, including the right coronary territory and collateral flow to the distal LAD [17]. Although Oh et al., Khan et al., and Matte et al. reported primary vessel occlusions of a hyperdominant‐LCx that jeopardized vast myocardium and yielded overt STE patterns, our cases involved selective ostial‐to‐proximal occlusion of the PDA branch alone. This more restricted infarction territory likely explains the subtle, nondiagnostic electrocardiographic findings in our patients, highlighting how the specific level of occlusion within a hyperdominant circuit dictates ECG visibility. To contextualize our findings, we performed a systematic literature review using PubMed, Scopus, and ScienceDirect to identify previously published cases of hyperdominant‐LCx. Pertinent demographic, clinical, angiographic, and therapeutic data were extracted and summarized in Table 3 [15, 18, 19, 20, 21, 22]. To the best of our knowledge, these represent the first documented cases of IPMI characterized by the unique combination of a left PDA arising from a hyperdominant‐LCx and nondiagnostic findings on standard 12‐lead ECG, a constellation that not only poses substantial initial diagnostic challenges but may also significantly affect patient outcomes.

**TABLE 3:** Case‐based review of hyperdominant left circumflex artery in the literature to date.

No.	Study type	Author and year	Age (years)/sex	Cardiovascular history/risk factors	Clinical presentation	Initial diagnosis	Standard 12‐lead ECG findings	Additional/posterior leads (V7–V9)	Peak troponin/cardiac biomarkers	Anatomical LCx variant and course	RCA and LAD morphology	Culprit vessel/segment	Revascularization/in‐hospital management	Clinical outcome/follow‐up
1	Images in cardiology	Canan and Batra, 2022 [18]	77/Female	HTN, T2DM	Syncope	Third‐degree AV block/Syncope (incidental SCA)	CHB	Not reported	Not reported	Hyperdominant‐LCx arising from a single left coronary artery, continuing into the right AV groove to supply the entire RCA territory, giving rise to SA nodal, PDA, and PLB branches	Absent RCA small LAD without obstructive disease	Not reported	PPM implantation (coronary anomaly managed conservatively as incidental finding)	Uneventful recovery; no recurrent syncope episodes reported at follow‐up
2	Case report and literature review	Oh et al. 2021 [15]	57/Male	Current smoker (30 pack‐years), Family history of premature CAD (father underwent CABG due to MI)	Chest pain (squeezing, without radiation for several hours), dyspnea, hypotension/shock (BP 70/40 mmHg), bilateral pulmonary crackles	AMI with STE (STEMI/posterior MI with cardiogenic shock)	STE in aVR; STD in precordial leads V2–V4	STE in leads V7–V9	hs‐cTnT: 0.020 ng/mL (Ref: 0–0.014); CK‐MB: 2.08 ng/mL (Ref: 0–5)	Hyperdominant‐LCx giving two large branches: one normal anterograde LCx branch and a large retrograde branch continuing into the AV groove supplying the entire RCA myocardium	Congenital agenesis of RCA; LAD with normal course without plaque	Proximal shaft of LCx (total occlusion/TIMI 0 flow)	Primary PCI to proximal LCx with predilatation (2.5 × 15 mm balloon at 6 atm), deployment of Sirolimus‐eluting stent, and post‐dilatation DAPT	Successful revascularization; Uneventful clinical recovery and hospital discharge
3	Case report	Rajlawot et al. 2023 [19]	70/Female	T2DM, HTN, and HLP; receiving medications for these conditions	Chest pain with associated shortness of breath relieved by rest and palpitations; positive treadmill test	Provisional diagnosis of stable angina at moderate exertion	ST‐segment changes; direction and lead distribution not reported	Not reported	Non‐elevated cardiac enzymes; assay, numerical values, and peak levels not reported	Hyperdominant‐LCx giving rise to the PDA, extending into the right AV groove and terminating near the right sinus of Valsalva to supply the RCA territory; described as a Lipton‐Yamanaka L‐I single‐coronary‐artery variant; eccentric calcified LCx plaques causing approximately 20%–30% luminal stenosis	RCA ostium and proximal segment absent on CCTA; left main arising normally from the left sinus of Valsalva and bifurcating into LAD and LCx; LAD diffusely diseased with approximately 70%–80% luminal stenosis	LAD (diffuse disease with approximately 70%–80% stenosis; treated vessel); no acute culprit lesion reported	Elective CAG followed by CCTA because the RCA was not adequately visualized; coronary calcium score > 800 Agatston units; subsequent LAD stenting and additional medical management for noncritical plaques; stent details and medications not reported	Not reported
4	Images in cardiology	Agrawal, 2015 [2]	56/Female	HTN; no other significant medical or surgical history reported	Unstable angina	Unstable angina with critical atherosclerotic stenoses of the LAD and first major diagonal branch (D1); incidentally identified hyperdominant‐LCx	Not reported	Not reported	Not reported	Extremely large, long, serpentine hyperdominant‐LCx giving rise to PDA and PLV branches and wrapping around the apex; supplies the apex and a significant proportion of the territories normally supplied by the RCA and LAD	RCA present, small‐caliber and nondominant; LAD small‐caliber, undersized Type I vessel, approximately 1.5–1.75 mm in diameter	LAD and D1: critical atherosclerotic stenoses; exact segments and a single culprit lesion not specified	Diagnostic invasive coronary angiography followed by PCI with stent deployment in the LAD and D1; stent specifications and adjunctive medical therapy NR	Asymptomatic after the procedure at the time of reporting; follow‐up duration NR
5	Case report	Choi et al. 2009 [20]	68/Female	Not reported	Occasional resting chest discomfort/atypical chest pain for 1 year; normal physical examination (normal BP, HR, and heart sounds); normal CXR, 2D echo, and Tc‐99m MIBI myocardial perfusion imaging	Single coronary artery with congenital absence of RCA and dominant LCx; suspected myocardial ischemia due to coronary slow flow phenomenon to the RCA territory without obstructive CAD or coronary spasm	Normal	Not reported	Normal (negative cardiac enzymes)	Large LCx coronary artery extending distally into the AV groove and continuing retrogradely as the RCA to supply the entire RCA territory, with abnormally slow angiographic filling; no coronary artery spasm on acetylcholine provocation	Absent RCA; LAD present without significant fixed stenosis	Not reported	CAG, aortography, and acetylcholine provocation test; conservative medical therapy with calcium channel blocker (Diltiazem) and nitrates	Resolution of atypical chest pain after medical therapy
6	Case Report	Iftikhar et al. 2019 [21]	45/Male	HTN, cigarette smoking	Chest pain and worsening shortness of breath over the last 3 days; severe LV systolic dysfunction on LV ventriculography	Anterior STEMI with severe LV systolic dysfunction	STE with Q waves in anterior leads	Not reported	Not reported	Hyperdominant‐LCx continuing beyond the crux into the right AV groove as an aberrant branch supplying the base of the heart and entire RCA territory, terminating near the right sinus of Valsalva; free of angiographic disease	Absent RCA; LAD with critical stenosis in its midcourse	Mid‐LAD	CAG, aortic root aortography, and LV ventriculography; primary PCI with stenting to mid‐LAD; advised myocardial perfusion imaging for myocardial viability assessment	Not reported
7	Case report	da Silva Matte et al. 2019 [17]	80/Male	HTN, hypothyroidism, previous smoking	Acute chest pain starting 5 h prior to admission; pulmonary basal rales on physical examination; post‐PCI severe LV systolic dysfunction (EF 26% on TTE)	Acute inferoposterolateral (STEMI) in the setting of acute hyperdominant‐LCx thrombosis with chronic LAD occlusion and congenital absence of RCA	Sinus rhythm with STE in inferior, lateral, and posterior leads	Posterior STE reported in conjunction with standard leads	Not reported	Hyperdominant‐LCx with high thrombotic subocclusion extending through the entire left AV groove into the right coronary topography, supplying right ventricular and atrial branches, as well as intracoronary collaterals to distal LAD	RCA absent; Proximal LAD chronically occluded	Proximal and mid LCx (acute subocclusive thrombus)	Emergency cardiac catheterization; primary PCI with deployment of 2 DESs to proximal and mid LCx after DAPT bolus; restoration of normal flow; conservative management of LAD CTO	Recovered well without major in‐hospital procedural complications; severe LV systolic dysfunction (LVEF 26% on TTE)

Abbreviations: 2D echo, two‐dimensional echocardiography; AMI, acute myocardial infarction; AV, atrioventricular; aVR, augmented voltage right arm; BP, blood pressure; CABG, coronary artery bypass grafting; CAD, coronary artery disease; CAG, coronary angiography; CCTA, coronary computed tomography angiography; CHB, complete heart block; CK‐MB, creatine kinase–myocardial band; CTO, chronic total occlusion; CXR, chest X‐ray; D1, first diagonal branch; DAPT, dual antiplatelet therapy; DES, drug‐eluting stent; ECG, electrocardiogram; EF, ejection fraction; HLP, hyperlipidemia; HR, heart rate; hs‐cTnT, high‐sensitivity cardiac troponin T; HTN, hypertension; LAD, left anterior descending artery; LCx, left circumflex artery; LV, left ventricular; LVEF, left ventricular ejection fraction; MI, myocardial infarction; NR, not reported; PCI, percutaneous coronary intervention; PDA, posterior descending artery; PLB, posterolateral branch; PPM, permanent pacemaker; RCA, right coronary artery; SA, sinoatrial; SCA, single coronary artery; STD, ST‐segment depression; STE, ST‐segment elevation; STEMI, ST‐segment elevation myocardial infarction; T2DM, Type 2 diabetes mellitus; Tc‐99m MIBI, technetium‐99m methoxyisobutylisonitrile (sestamibi); TIMI, thrombolysis in myocardial infarction; TTE, transthoracic echocardiography.

Despite the absence of clearly defined guideline‐based criteria for the use of additional diagnostic leads, in accordance with the same ACS guidelines, both patients were classified as very‐high risk NSTEMI and were therefore immediately referred for coronary catheterization. Although echocardiographic correlation with cardiac magnetic resonance imaging might have demonstrated segmental wall‐motion abnormalities that are typically observed in IPMI, our emergency physicians did not perform pre‐procedural imaging because neither patient exhibited guideline‐recommended indications, a diagnostic approach that could have further extended the first medical contact (FMC)–to–balloon time [8, 9]. Therefore, in these cases, the diagnosis of IPMI was established retrospectively by CAG findings.

Indeed, IPMI has been only limitedly discussed in earlier studies [6]. Shimojo et al. discussed that posterior leads (V7–V9) aid in diagnosing PMI and may improve prognostic assessment [23]. A case report has described that LCx‐related infarctions are often hidden on standard 12‐lead ECG and may require additional leads for accurate detection [12]. To identify reported cases, we conducted a systematic literature search (2000–present) across PubMed, Scopus, ScienceDirect, and Google Scholar. The search strategy utilized Boolean operators (AND/OR) to combine MeSH/entry terms, synonyms, and abbreviations for: “Myocardial Infarction,” “Posterior myocardial infarction,” “Isolated posterior myocardial infarction,” “Acute Coronary Syndrome,” “Electrocardiography,” “Posterior leads,” “Right coronary artery,” “Posterior descending artery,” “Left circumflex artery,” “Left anterior descending artery,” and “Left main coronary arter.” Moreover, a manual search of the reference lists of all included studies and relevant case reports was conducted to detect any additional eligible studies. In total, we found 13 papers, which included 15 cases (Table 4). There are nine males and six females, with an average age of 57.61 ± 10.11 years. In five cases, the initial diagnosis was NSTEMI, and in nearly all cases, 12‐lead ECG changes were observed in the anterior precordial leads (V1–V4). To comprehensively review the most relevant original studies on IPMI, we performed a literature search using synonymous keywords across established scientific databases (Table 5). Eleven relevant studies were found, comprising a total of 136,160 patients, of which only 970 patients (~0.7%) were diagnosed with IPMI, underscoring the rarity of this condition. Importantly, a pattern observed across reviewed studies was the persistent diagnostic challenge of IPMI and its frequent missed diagnosis in clinical practice. Crucially, our literature review revealed that the vast majority of patients exhibited STD across the conventional anterior precordial leads (V1–V4). Rather than being misinterpreted as primary anterior subendocardial ischemia, this critical finding highlights that such precordial changes typically serve as the reciprocal “mirror image” manifestation of true posterior transmural injury. This observation underscores, more than ever, the vital necessity of promptly obtaining additional posterior leads (V7–V9) whenever suspicious precordial depression is encountered, thereby unmasking otherwise concealed occlusion MIs [22, 23, 24, 25, 26, 27, 28, 29, 30, 31, 32, 33, 34, 35, 36, 37, 38, 39, 40, 41, 42]. A pivotal insight emerging from this review is that, although the occlusive/nonocclusive paradigm has substantially improved the detection of true acute coronary occlusion beyond the conventional STEMI/NSTEMI criteria, particularly in posterior wall infarction, where ischemic STD in leads V1–V4 serves as a highly specific marker (specificity ∼97%), as frequently documented across the literature; however, both of our cases exhibited a distinct absence of these hallmarks. Therefore, our findings suggest that hyperdominant‐LCx anatomy may represent a particularly challenging substrate in which even established occlusive myocardial infarction‐associated ECG markers are absent, contributing to the under‐recognition of IPMI and highlighting its multifaceted diagnostic challenges [43, 44]. The results of this literature review are not generalizable to the broader population [22, 23, 24, 25, 26, 27, 28, 29, 30, 31, 32, 33, 34, 35, 36, 37, 38, 39, 40, 41, 42]. Although beyond challenging conventional diagnostic paradigms, future practice guidelines should incorporate more refined criteria and precise clinical triggers for the early detection of IPMI (Figure 7).

**TABLE 4:** Literature review of IPMI cases.

Number	Type of study	Author and year	Sex	Age (year)	Prior heart disease	Coronary dominant	Initial diagnosis	Presenting symptoms	ECG findings	Troponin‐T values	Investigations	Treatments	Additional leads findings
1	Case report	Wijaya et al. 2025 [24]	Male	56	No	Not reported	STEMI	1. Atypical angina chest pain 2. Serve back pain 3. Cold sweating	STD in V1–V4	Not performed	Not performed	Medical fibrinolytic therapy	Recent STE, T wave inversion, and pathologic Q wave in V7–V9
2	Case report	Moskovitz et al. 2025 [25]	Male	58	Yes	Right	NSTEMI	Substernal chest pain radiating to his arms	1. STD in leads V2–V4 2. Poor R progression	Not reported	Triple‐vessel disease with total occlusion in the LCx	Two DESs in the middle segment of LCx	Normal
3	Case report	Rakun and Yeo 2025 [26]	Male	48	NO	Right	STEMI	Isolated left arm numbness with no chest pain (painless)	STD and T wave inversions in leads V2–V5	8518 ng/L	Total thrombotic occlusion of the proximal LCx	Coadjutant pharmacologic therapy DES the proximal segment of LCx	STE in leads V7–V9
4	Case report	García‐Arias et al. 2024 [14]	Female	47	No	Right	NSTEMI	Chest pain radiating to both arms, associated with nausea and diaphoresis	1. Symmetrical T‐wave inversion 2. Down‐sloping STD 3. High R waves in V1–V3	432 ng/L	Total thrombotic occlusion of the proximal LCx	Coadjutant pharmacologic therapy DES in the D1 branch	STE in leads V7–V9
5	Images in cardiology	Macherey‐Meyer et al. 2023 [27]	Female	61	No	Not reported	STEMI	Typical anginal chest pain	STD in V1–V3	240 ng/L	Triple‐vessel disease with total occlusion of the LCx	Complete Revascularization in the LCx	STE in leads V7–V9
6	Case report	Komminen et al. 2023 [28]	Male	45	NO	Not reported	NSTEMI	1. Substernal, non‐radiating, pressure‐like chest pain 2. Nausea and dizziness 3. Ongoing shortness of breath with little exertion	1. STD in V2–V4 2. T wave inversions in V1–V4	Not reported	Significant myocardial bridging in the mid to distal LAD without evidence of stenosis	Discharged with an outpatient cardiac MR	Not performed (missed)
7	Case series	Alsagaff et al. 2022 [6]	Male	57	No	Right	STEMI	Typical anginal chest pain + dyspnea	STD in leads V2–V4	5413 ng/L	Triple‐vessel disease with total occlusion of the LCx	DES in the LCx + Adjutant pharmacologic therapy	STE in leads V7–V9
8	Case series	Alsagaff et al. 2022 [6]	Male	62	No	Right	NSTEMI	Typical anginal chest pain	STD in V1–V3	4068 ng/L	Two‐vessel disease with total occlusion of the LCx	Complete revascularization in the LCx and LAD	Not performed (missed)
9	Case Series	Alsagaff et al. 2022 [6]	Male	53	No	Right	STEMI	Referred chest pain	Iso‐electric ST segment in V1–V3	13,061 ng/L	Two‐vessel disease with total occlusion of the LCx	Complete revascularization in the LCx and LAD	STE in leads V7–V9
10	Case report	Pratama et al. 2022 [7]	Female	45	No	Right	STEMI	1. Atypical anginal chest pain 2. Burning sensation in the left chest 3. Radiation pain to both arms, legs, and back	STD in V1–V4	4370 ng/L	Total occlusion of the first diagonal branch (D1) of the LAD	DES in the D1 branch+ Adjutant pharmacologic therapy	1. STE in leads V7–V9 2. Occasional PVCs
11	Case report	Waddell et al. 2022 [29]	Female	74	No	Right	STEMI	1. Worsening of her chronic reflux disease 2. Two‐day history of waxing and waning substernal discomfort	1. Tall R waves in V2 2. Poor R‐wave progression 3. Upright T waves 4. STD in V2–V3	20,694.5 ng/mL	Triple‐vessel disease with severe stenosis in the LCx	Not reported	STE of more than 0.5 mm in leads V7 and V8
12	Case report	Ramjaun et al. 2021 [30]	Male	70	Not reported	Not reported	NSTEMI	Chest pain and fatigue	STD in V2–V4	330 ng/L	Triple‐vessel disease with total occlusion in the LCx	DES in the LCx	Normal
13	Case report	Hähnle et al. 2020 [31]	Female	65	No	Right	STEMI	Severe central crushing chest pain associated with autonomic symptoms	STD in leads V1–V4	Not reported	Triple‐vessel disease with total occlusion of the LCx	DES in D1 branch+ Adjutant pharmacologic therapy	STE in leads V7–V9
14	Case report	Cornejo‐Guerra et al. 2018 [32]	Male	74	No	Not reported	STEMI	Substernal chest pain	1. Broad R waves 2. ST‐segment down‐sloping in right precordial leads	2500 ng/L	Total occlusion in the proximal segment of LCx	DES in the LCx	STE and pathological Q waves in leads V7–V9
15	Images in cardiology	Zakaria et al. 2012 [33]	Female	69	No	Not reported	STEMI	1. Substernal chest pain 2. Progressive dyspnea	STD with upright T waves in leads V3–V4	Not reported	Total occlusion in the proximal segment of LCx	DES in the LCx	STE in leads V7–V9

Abbreviations: DES, drug‐eluting stent; LAD, left anterior descending artery; LCx, left circumflex artery; NSTEMI, non‐ST‐segment elevation myocardial infarction; STD, St‐segment depression; STEMI, ST‐segment elevation myocardial infarction.

**TABLE 5:** Literature review of original research on IPMI.

Number	Authors and year	Study population	Patients with isolated V7–9 STEMI	What was found?
1	Boden et al. 1987 [34]	In patients with creatine kinase (CK)‐MB confirmed non‐Q‐wave AMI randomized to the prospective multicenter Diltiazem Reinfarction Study	23	In a multicenter prospective study of 544 patients with biochemically confirmed non‑Q‑wave AMI, 50 patients (9.2%) demonstrated isolated precordial STD ≥ 1 mm in at least two leads (V1–V4). Serial ECG evaluation revealed that 23 of these patients (40%) later developed ECG evidence of PMI, defined by an R wave ≥ 0.04 s in V1 and an R/S ratio > 1 in V2, predominantly by day 3 (78%). Patients who progressed IPMI exhibited greater horizontal STD with upright T waves, markedly higher peak CK levels, and more pronounced STD in V1–V3 compared with patients who retained features of anterior non‑Q‑wave AMI.
2	Roul et al. 1991 [35]	In patient with primary MI	28	In a prospective study of 328 patients with first‑time MI, isolated STD in leads V2–V4 was identified in 28 patients (8.5%). The STD was most pronounced in lead V3 (1.8 ± 0.7 mm) or lead V4 (2.0 ± 1.0 mm), with persistently positive T waves. All affected patients demonstrated posterolateral left ventricular wall‐motion abnormalities on two‐dimensional echocardiography. Pathological Q waves in leads V7–V9, along with an increased R/S ratio in leads V1–V2, appeared at a mean of 33 h after symptom onset (range: 10–56 h). CAG, performed in 26 patients, revealed significant LCx disease in all cases, either as isolated involvement (39%) or in association with LAD (15%), RCA disease (19%), or both (27%).
3	Casas et al. 1997 [36]	All hospitalized patients who had 12‑lead ECGs during an 18‑month period	27	In 17,000 consecutive hospitalized patients underwent 12‑lead ECG over 18 months, 250 patients had posterior leads (V7–V9) recorded due to tall R waves in V1–V2 or suspicion of IPMI. PMI was detected in 110 patients by STE and/or broad Q waves in V7–V9, with 27 cases diagnosed solely by posterior leads and 83 showing additional inferior or lateral involvement. In the remaining 140 patients, posterior leads were normal. Conditions associated with tall R waves in V1–V2, including RBBB, right ventricular enlargement, and WPW syndrome, did not produce diagnostic changes in V7–V9, supporting the diagnostic value of routine posterior lead recording.
4	Matetzky et al. 1999 [37]	Consecutive patients with ischemic chest pain	33	In this study, 33 consecutive patients presenting with ischemic chest pain consistent with AMI were enrolled. None of the patients demonstrated STE on the standard 12‑lead ECG; however, STE was identified in the posterior leads V7–V9. Among these patients, STD in leads V1–V3 was observed in 20 individuals (61%). In addition, pathological Q waves in leads V7–V9 were present in 75% of patients. On echocardiography, posterior wall‐motion abnormality was visible in 97% of the patients, and 69% had evidence of MR, which was moderate or severe in one‐third of the patients CAG, performed in 20 patients, identified the LCx as the infarct‐related artery in all cases.
5	Oraii et al. 1999 [38]	In consecutive patients with AMI at the CCU	7	In 210 consecutive patients with AMI, posterior ECG leads (V7–V9) recorded at presentation. STE ≥ 1 mm in ≥ 2 posterior leads was identified in 19 patients (9%), occurring alone in seven cases (3.3%) or with inferior or lateral STE in 12 cases (5.7%). Posterior STE was detected in 10.9% of patients without STE on standard ECG, and tall R waves in V1–V2 developed in 26.3%. In‐hospital complications were significantly more frequent in the IPMI group compared with matched controls.
6	Agarwal et al. 1999 [22]	Only for patients without 12‐lead STEMI	18	Posterior pericordial leads V7–V9 were recorded among 58 patients suspected MI and a nondiagnostic standard ECG. STE > 0.1 mV or Q waves in ≥ 2 posterior leads were predefined as diagnostic criteria for IPMI. Eighteen patients met ECG criteria for posterior MI. All 18 cases were later confirmed to have MI based on creatine phosphokinase elevation and/or CAG. Of the 17 patients who underwent CAG, 16 had LCx involvement as the culprit vessel. Door‐to‐balloon times were prolonged among patients with IPMI (107 min vs. 72 min), a smaller proportion achieved reperfusion within 90 min.
7	Wung et al. 2001 [39]	In patients who underwent elective angiography for LCx	While STE confined to the posterior leads (phenotypically similar to the ECG pattern of IPMI) was observed in five patients, this study based on a controlled, reversible balloon‑occlusion ischemia model, and no patients developed true IPMI.	In this study, 15‑lead ECGs (12 standard leads plus V7–V9) were obtained in 53 patients underwent elective LCx angioplasty to represent posterior ischemia. During balloon occlusion, posterior STE was frequently observed, and 9% of patients had IPMI detected only in posterior leads, which was not detected on the standard 12‑lead ECG. With a 1‑mm STE cutoff, the sensitivity of the 15‑lead ECG was similar to the 12‑lead ECG. Notably reducing the posterior lead threshold to 0.5 mm markedly increased sensitivity to 94%, suggesting that posterior leads are diagnostically useful only when a lower STE criterion is applied
8	Aqel et al. 2009 [40]	In patients who underwent clinically indicated coronary interventions	In this study, patients were subjected to a controlled ischemia model induced by temporary balloon occlusion of the LCx artery; consequently, no cases of IPMI.	In this study, 53 patients undergoing coronary intervention were assessed to examine whether adding posterior ECG leads (V7–V9) improves detection of LCx‑related ischemia. Posterior ischemia was produced by temporary balloon occlusion of the LCx. During occlusion, STE was greater and more commonly detected in posterior leads than in the standard 12‑lead ECG. The 15‑lead ECG identified STE in ≥ 2 contiguous leads more often than the 12‑lead ECG at both > 0.5 mm and > 1 mm thresholds. Adding posterior leads therefore improves detection of IPMI and may aid more timely diagnosis and reperfusion therapy
9	Khan et al. 2012 [41]	This study was designed to recognized the ability of physicians and healthcare personnel in diagnosing IPMI	Not reported	In this study, doctors and paramedics were asked to assess a 12‑lead ECG consistent with posterior ischemia and were tested on their ability to detect IPMI and the need for posterior leads. Among 117 doctors, only 38% recognized IPMI as a potential diagnosis, with recognition increased with level of training. Only 50% of those who suspected IPMI recognized the need for posterior leads for confirmation, and fewer than 19% were able to correctly position the leads or identify the diagnostic criterion (STE ≥ 1 mm in V7–V9). Among nurses, 22% correctly positioned posterior leads, while only 10% of paramedics identified IPMI. In general, both doctors and paramedics were significantly more accurate in diagnosing anterolateral MI than IPMI
10	Waldo et al. 2014 [42]	In patients with an IPMI and those with all other STEMI	824	In this study individuals with IPMI were compared with those with other STEMI, assessing reperfusion times and risk adjusted in hospital mortality. Among 117,739 STEMI patients, 824 (0.7%) had IPMI. Arrival‑to‑ECG time did not differ between groups (6 vs. 6 min). However, IPMI patients had longer ECG‑to‑PCI times (69 vs. 61 min) and were less likely to meet door‑to‑balloon < 90 min (83% vs. 89%). After multivariable adjustment, in‑hospital mortality was similar between IPMI and non‑IPMI STEMI patients
11	Shimojo et al. 2022 [23]		10	In this study, 142 STEMI patients with a LCx culprit lesion were analyzed. Ten patients (7.0%) demonstrated STE only in synthesized posterior leads V7–V9, while the remaining 132 patients (93%) presented with STE on the standard 12‑lead ECG. Patients with STEMI detected only in synthesized V7–V9 leads experienced higher rates of cardiac mortality at both 3 months and 1 year (30% vs. 6.1% and 30% vs. 7.6%, respectively) as well as a higher incidence of mechanical complications (20% vs. 1.5%) compared with patients diagnosed on the standard 12‑lead ECG.

Abbreviations: AMI, acute myocardial infarction; CAG, coronary angiography; CK_MB, creatine kinase—myocardial band; CPK, creatine phosphokinase; ECG, electrocardiogram; IPMI, isolated posterior myocardial infarction; LAD, left anterior descending artery; LCx, left coronary circumflex artery; MI, myocardial infarction; MR, mitral regurgitation; PMI, posterior myocardial infarction; RBBB, right bandle branch block; RCA, right coronary aster; STD, ST‐segment depression; STE, ST‐segment elevation; STEMI, ST‐segment myocardial infarction; WPW, Wolf‐Parkinson‐White.

**FIGURE 7:**
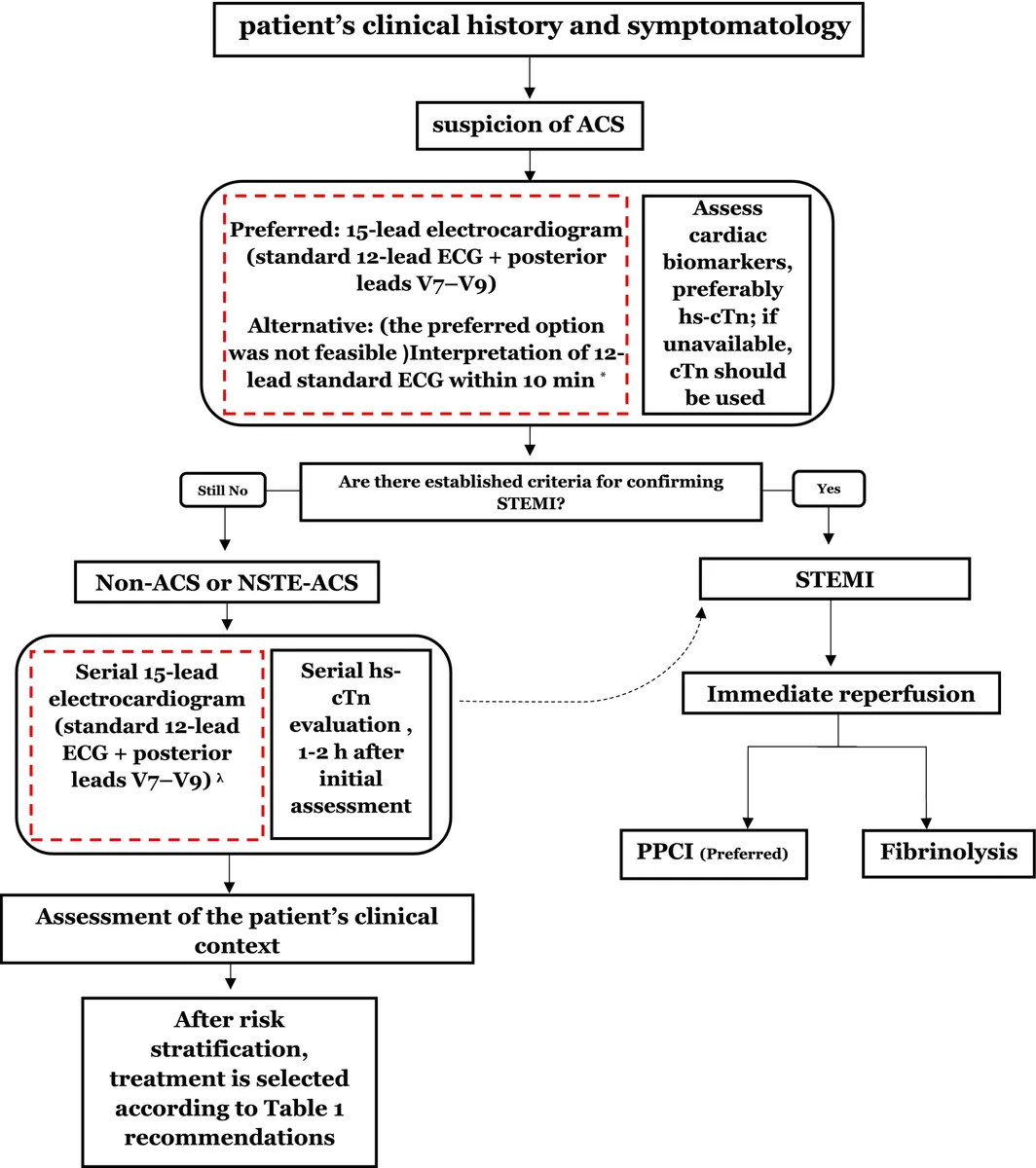
A suggested diagnostic approach based on our clinical experience to prevent overlooking concealed IPMI particularly in left‐dominant coronary circulation. *We suggested that performing an initial 15‐lead ECG, including posterior leads in all symptomatic patients with suspected ACS; however, this may not always be feasible at the FMC. ^λ^At this point, we recommend recording a 15‐lead ECG, including posterior leads (V7–V9) within 90 min of the initial ECG, in all patients.

PCI remains the preferred treatment of reperfusion for patients with IPMI. These patients typically experience a prolonged interval from first medical contact (FMC) to PCI, with fewer achieving door‐to‐balloon times < 90 min [6, 8]. In addition, when left‐dominant coronary anatomy is present, it is generally associated with worse outcomes [1, 8]. Early reperfusion of the infarct‐related artery improves myocardial salvage, increases survival rates, and reduces adverse clinical outcomes [8, 12]. In both of our cases, early intervention led to favorable short‐term recovery and sustained favorable outcomes at follow‐up to date.

## 5 Limitations

Several inherent limitations should be noted. First, the non‐systematic review approach carries potential selection bias without ensuring comprehensive evidence capture. Second, a two‐case series inherently precludes establishing causal or prognostic links between hyperdominant‐LCx anatomy and IPMI. Third, drug‐coated balloons were not accessible at our catheterization laboratory despite their established clinical utility. Fourth, non‐culprit lesions, including intermediate LAD/RCA stenoses in the first patient and an OM1 lesion in the second, could not undergo physiological interrogation (fractional flow reserve [FFR]/instantaneous wave‐free ratio [iFR]) due to institutional resource constraints. Finally, hyperdominant‐LCx lacks a universally accepted definition, relying predominantly on heterogeneous case reports; future large‐scale angiographic cohorts are warranted to standardize diagnostic criteria and define its prevalence and clinical significance.

## 6 Conclusion

Diagnosing ACS with a hyperdominant LCx is clinically challenging and often delays intervention. Our cases demonstrate that relying on conventional posterior leads may fail to identify IPMI in specific anatomical variants. The limited sensitivity of standard ECG (50%–60%) and STEMI criteria (~40%) for occlusive myocardial infarction, combined with a specificity (~96%) inherently biased toward V1–V4 leads, creates a diagnostic blind spot that effectively masks variant forms of IPMI. Consequently, IPMI may manifest only with subtle, nondiagnostic alterations or complete electrical concealment in standard 12‐lead ECGs. Furthermore, current guideline‐based criteria serve as a double‐edged sword in rare coronary variants, potentially precipitating missed diagnoses, yet conversely enabling prompt reperfusion when appropriately contextualized.

## Author Contributions

**Amirmahdi Mohammadi:** conceptualization, investigation, funding acquisition, writing – original draft, writing – review and editing, visualization, validation, methodology, project administration, formal analysis, software, resources, supervision, data curation. **Mohammad Sadegh‐Vashveshady:** conceptualization, investigation, supervision.

## Funding

The authors have nothing to report.

## Ethics Statement

This study was approved by the Research Ethics Committee of Semnan University of Medical Sciences and Health Services, Semnan, Iran (Ethics code: IR.SEMUMS.REC.1404.249).

## Consent

Written informed consent was obtained from all patients for publication of their clinical details and images in line with COPE guidance.

## Conflicts of Interest

The authors declare no conflicts of interest.

## Data Availability

Data sharing is not applicable to this article as no datasets were generated or analyzed during this study.
